# Evidence of structural invariance across three groups of Meehlian schizotypes

**DOI:** 10.1038/npjschz.2016.16

**Published:** 2016-05-04

**Authors:** Raymond CK Chan, Diane C Gooding, Hai-song Shi, Fu-lei Geng, Dong-jie Xie, Zhuo-Ya Yang, Wen-hua Liu, Yi Wang, Chao Yan, Chuan Shi, Simon SY Lui, Eric FC Cheung

**Affiliations:** 1 Neuropsychology and Applied Cognitive Neuroscience Laboratory, Key Laboratory of Mental Health, Institute of Psychology, Chinese Academy of Sciences, Beijing, China; 2 Department of Psychology, University of Wisconsin-Madison, Madison, WI, USA; 3 Department of Psychiatry, University of Wisconsin-Madison, Madison, WI, USA; 4 The University of Chinese Academy of Sciences, Beijing, China; 5 Faculty of Health Management, Guangzhou Medical University, Guangzhou, China; 6 Key Laboratory of Brain Functional Genomics (MOE & STCSM), School of Psychology and Cognitive Science, East China Normal University, Shanghai, China; 7 Peking University Sixth Hospital, Beijing, China; 8 Peking University Institute of Mental Health, Beijing, China; 9 Department of General Adult Psychiatry, Castle Peak Hospital, Hong Kong Special Administrative Region, Hong Kong, China

## Abstract

According to Meehl’s model of schizotypy, there is a latent personality organization associated with the diathesis for schizophrenia that can be identified in several ways. We sought to examine the structural invariance of four Chapman psychosis–proneness scales (CPPS) across three groups of putative schizotypes, namely, clinically-, biologically-, and psychometrically-identified schizotypes. We examined the factor structure of the Perceptual Aberration (PER), Magical Ideation (MIS), Revised Social Anhedonia (RSAS), and Revised Physical Anhedonia (RPAS) scales in 196 schizophrenia patients, 197 non-psychotic first-degree relatives, and 1,724 non-clinical young adults. The confirmatory factor analyses indicated that the best-fitting model was one in which there is a two-factor model with negative schizotypy (RSAS and RPAS) and positive schizotypy (PER and MIS). All three samples fit the model well, with Comparative Fit Indices>0.95 and Tucker Lewis Indices>0.90. The root mean square error of approximations were all small (*P* values⩽0.01). We also observed that for both anhedonia scales, the groups’ mean scale scores varied in the hypothesized direction, as predicted by Meehl’s model of schizotypy. All three Chinese samples, namely, the patients (clinical schizotypes), relatives (biologically-identified schizotypes), and non-clinical young adults (containing psychometrically-identified schizotypes) showed the same factorial structure. This finding supports the suitability of the CPPS for cross-cultural and/or genetic investigations of schizotypy.

## Introduction

Identifying traits that genetically correlate with schizophrenia is an important strategy for detecting genes that confer risk for schizophrenia.^1,2^ In his seminal paper on the etiological basis of schizophrenia, Meehl^3^ posited the existence of subclinical schizophrenia-like traits that are manifestations of an inherited genetic diathesis and environmental experiences. The resultant syndrome is a latent personality organization that he termed “schizotypy”. According to Meehl,^3,4^ these schizophrenia-like traits included anhedonia, interpersonal aversiveness, ambivalence, and cognitive slippage. Individuals with schizotypy are referred to as “schizotypes”. Given the principle of multifinality, schizotypy has multiple possible outcomes, including: clinical conditions genetically related to schizophrenia, including schizotypal personality disorder; aberrant performance on putative endophenotypic measures of a genetic diathesis, such as smooth pursuit eye tracking,^1,5^ working memory,^1,6^ and cognitive slippage;^7,8^ and deviant performance on psychometric measures of schizotypy. A subset of individuals with schizotypy was theorized to develop the full clinical presentation of schizophrenia.

Due to their genetic relatedness to individuals with schizophrenia, first-degree biological relatives of schizophrenia patients are more likely to possess schizotypy, the latent personality organization associated with a genetic diathesis for schizophrenia, than other individuals in the general population. Although not all first-degree relatives possess schizotypy, studying these individuals is a viable strategy to investigate the schizophrenia diathesis. The Chapmans and their students developed their psychosis–proneness scales to psychometrically detect such schizotypal individuals in the Meehlian sense.

Several independent researchers^9,10^ have since demonstrated that schizotypy can be detected in the general population using these reliable, validated psychometric measures. For example, Gooding *et al.*^10^ demonstrated that some individuals who possess schizotypy could be identified on the basis of their psychometric profiles, despite not differing from their comparison peers in terms of family histories of psychotic illness. Thus, in addition to identifying schizotypes on the basis of their biological relatedness to a person with schizophrenia, or on the basis of their clinical symptomatology, another strategy is to identify such individuals using psychometric means.

Some investigators^2,11^ have asserted that questionnaires may be less sensitive to schizotypal traits in relatives of schizophrenia patients, compared with interview-based measures. However, interview-based measures are subject to methodological problems, such as interrater reliability and interviewer expectations, which may inadvertently affect ratings. Indeed, there is evidence that use of questionnaire methods may be preferable in some cases, particularly when inquiring about stigmatizing conditions such as psychiatric illness.^12^ The purpose of the present investigation was to examine whether there is evidence of structural invariance in the Chapman psychosis–proneness scales (CPPS), which are perhaps one of the most widely used questionnaire measures in schizotypy research.

Previously, we^13^ found evidence of measurement invariance of the CPPS across culture and time. We now wish to explore the ability of the CPPS to measure schizopphrenia-like traits across the entire spectrum of Meehlian schizotypy. According to Meehl’s model, all the schizophrenia patients are expected to have relatively high degrees of schizotypy, whereas the first-degree relatives would be characterized by varying degrees of schizotypy. In contrast, a subset (perhaps 10%) of the non-clinical samples could also be expected to possess some degree of schizotypy. We hypothesized that schizophrenia patients would have higher scores on the CPPS than their non-psychotic first-degree relatives, who in turn, were expected to have higher mean scores than participants in a non-clinical sample. Despite the differences in score magnitude, which could in part reflect differences in terms of the expected proportion of schizotypy in each group, we hypothesized that the CPPS would display relative structural invariance across the three groups.

## Results

Table 1 summarizes the demographic information and the scale scores on the CPPS for each of the samples. We observed impressive internal consistencies for all the scales in patients with schizophrenia (Cronbach *α* ranging from 0.83 to 0.93), non-psychotic first-degree relatives (Cronbach *α* ranging from 0.82 to 0.94), and students (Cronbach *α* ranging from 0.75 to 0.89). After parceling, the scales’ reliabilities ranged from 0.78 to 0.93 in patients, from 0.79 to 0.94 in non-psychotic first-degree relatives, and from 0.77 to 0.89 in college students. The reliabilities of three samples before and after parceling are shown in Supplementary Table 1.

**Figure 1:**
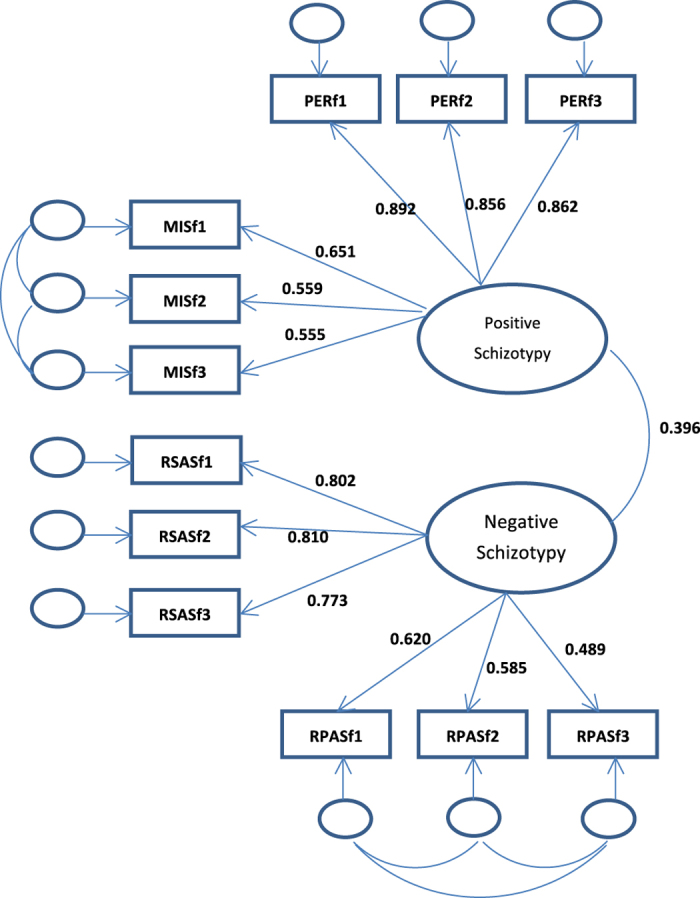
This is the two-factor model with the items of the Revised Social Anhedonia (RSAS) and the Perceptual Aberration (PER) scales independent and two factors, namely, positive schizotypy and negative schizotypy, correlated. Note: PERf1, PERf2, and PERf3 refer to the three parcels of the PER scale; RSASf1, RSASf2, and RSASf3 refer to the three parcels of the RSAS scale. This figure shows the results of a confirmatory factor analysis, whereby the three parcels of the Magical Ideation Scale (MIS) and PER scales load on the positive schizotypy factor, and the three parcels of the RSAS and the three parcels of the Revised Physical Anhedonia Scale (RPAS) load on the negative schizotypy factor. This two-factor model corresponds with the original theory underlying the construction of the Chapman psychosis–proneness scales. This figure also shows the standardized coefficients for the best-fitting model. The correlation coefficient is 0.396. Reprinted from Psychiatry Research, vol. 228, issue 1, Chan, R.C.K., Shi, H.-s., Geng, F.-l., Liu, W.-h., Yan, C., Wang, Y., & Gooding, D.C., “The Chapman psychosis-proneness scales: Consistency across culture and time”, pp. 143- 149, copyright 2015, with permission from Elsevier.

We used the best-fitting model in Chinese samples,^13^ as the default model for testing. The model is provided in Figure 1, in which parameters were calculated using all the participants. In an earlier investigation,^13^ we tested a unidimensional model, in which all the variables loaded onto one schizotypy factor, as well as a two-dimensional model in which social anhedonia loaded on both the positive and negative schizotypy factors. We previously found that the best-fitting model is a two-factor model with positive schizotypy (Magical Ideation Scale (MIS) and Perceptual Aberration (PER) subscales) and negative schizotypy (Revised Social Anhedonia Scale (RSAS) and Revised Physical Anhedonia Scale (RPAS) subscales) as correlated but independent dimensions.

**Figure 2:**
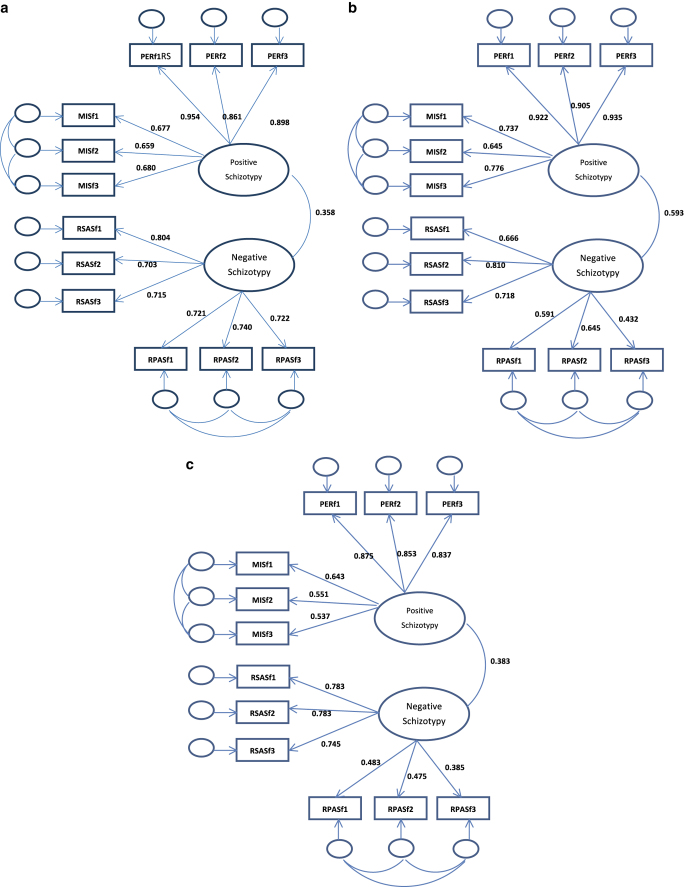
(**a**) This is the two-factor model with the items of the Revised Social Anhedonia (RSAS) and the Perceptual Aberration (PER) scales independent and two factors, namely, positive schizotypy and negative schizotypy, correlated in schizophrenia patients. Note: PERf1, PERf2, and PERf3 refer to the three parcels of the PER scale; RSASf1, RSASf2, and RSASf3 refer to the three parcels of the RSAS scale. This figure shows the results of a confirmatory factor analysis, whereby the three parcels of the Magical Ideation Scale (MIS) and PER scales load on the positive schizotypy factor, and the three parcels of the RSAS and the three parcels of the Revised Physical Anhedonia Scale (RPAS) load on the negative schizotypy factor. This two-factor model corresponds with the original theory underlying the construction of the Chapman psychosis–proneness scales. The correlation coefficient between the positive schizotypy and negative schizotypy factors is 0.36. (**b**) This is the two-factor model with the items of the RSAS and the PER scales independent and two factors correlated in first-degree relatives of schizophrenia patients. The correlation coefficient between the positive schizotypy and negative schizotypy factors is 0.59. (**c**) This is the two-factor model with the items of the RSAS and the PER scales independent and two factors correlated in non-clinical young adults. The correlation coefficient between the positive schizotypy and negative schizotypy factors is 0.38.

We tested the models in all three samples independently, and the results are shown in Table 2. Various goodness-of-fit indices indicated that the two-factor model of schizotypy showed a good fit to the data in each of the three samples, with Comparative Fit Index (CFI)>0.95, Tucker–Lewis index (TLI)>0.90, and Standardized Root Mean Square Residual (SRMR) values all acceptable, and less than 0.08.^14^ Figures 2a–c show the models with standardized coefficients in each of the groups.

The results of the cross-sample invariance test are provided in Table 3. For testing the measurement invariance, we tested for form invariance first, then constrained the lambdas to be equal across samples, and then constrained the indicators‘ intercepts equal further, and then constrained the error variance equal. For testing for structure invariance, we constrained the factors’ variance and covariance to be equal across groups, and then constrained the factor means to be equal.

For testing measurement invariance, we tested for form (or configural) invariance first (Model 1). We then constrained the factor loadings to be equal across samples (Model 2) to test for metric invariance. We looked for evidence of scalar (or strong factorial) invariance by comparing the latent means, and constraining the item intercepts to be the same across the groups (Models 3 and 5) and then constrained the error variances to equal to test for strict factorial invariance (Models 4 and 6). For testing for structure invariance, we constrained the factors’ variance and covariance to be equal across groups (Model 7), and then constrained the factor means to be equal (Model 8.)

The results showed that when constraining the factor loadings to equal across samples, the fitness of the model changed slightly. We used the fitness indicator difference test because our sample was too large for the *χ*^2^-tests to be insignificant.^15,16^ When the difference of the fitness indicator is <0.01, it indicates no significant difference. When the difference is between 0.01 and 0.02, it indicates there is a slight difference. If the difference of the fitness is >0.02, it indicates a significant difference. As seen in Table 3, the model has partial Metric (or weak) invariance across schizophrenia patients, relatives, and college students. That is, the data suggest that across the different groups, the strengths of the associations between specific scale items and their respective latent constructs are the same. However, we noted that the items’ intercepts and error variances were different in the three groups. We found structure invariance only at the factor variance and covariance level; the factor means were different between the groups. This suggests that all latent variables have the same relationship in the three groups of schizotypes, though the groups may differ in the extent to which they are characterized by the latent variable.

The between-group comparison of mean CPPS scores is provided in Table 1.

Schizophrenia patients reported significantly higher PER scores than both the relatives (*P*<0.01) and the college students (*P*<0.001), although the latter two groups did not differ from each other. The college students reported significantly greater magical thinking than the patients (*P*<0.01), who in turn had higher MIS scores than the relatives (*P*<0.01). On both physical and social anhedonia scales, schizophrenia patients scored significantly higher than both the relatives (*P* values<0.001) and the college students (*P* values<0.001).

## Discussion

To our knowledge, the present investigation includes the largest sample of schizophrenia patients and first-degree relatives using the CPPS to date. Using confirmatory factory analysis, we found, in a non-Western culture, that the best fit model for schizotypy was a two-factor model with positive and negative schizotypy. This two-factor model was consistent across a schizophrenia patient sample, relative sample, and a sample of Chinese young adults. Our findings provide ample support that the CPPS perform similarly, i.e., are able to detect the presence of schizotypal traits, across a continuum of scores and samples.

These findings extend the extant literature, and suggest that the CPPS are valid and robust in Chinese samples, as well as in American and European samples. In first-episode schizophrenia,^17^ as well as more chronic patients,^18,19^ PER scores have been found to be higher than those self-reported by first-degree relatives (siblings or parents). Our results are consistent with these previous reports. To date, there is relatively little comparative data regarding MIS scores of relatives of schizophrenia patients. However, we observed significantly greater magical thinking among the young adults than either the patient group or the relatives. Replication is necessary to discern whether this finding reflects a cohort effect, a defensive test-taking attitude, or a true difference in schizotypal traits. Given comparisons with other non-clinical student samples, however, we are inclined to regard this finding as a cohort effect.

We were especially interested to observe that both the RSAS and RPAS scores followed a linear pattern, in which patients had the highest mean scores, their relatives had intermediate scores, and the non-clinical students had the lowest scores. These findings were in general consistent with our previous findings across the Chinese samples of schizophrenia patients, schizotypy, and non-schizotypy groups,^20^ and wholly consistent with those of Kuha *et al.,*^21^ whose Finnish sample of schizophrenia-spectrum probands displayed significantly higher mean RSAS and RPAS scores than their unaffected siblings. Our present findings, particularly the rather robust difference in mean RSAS scores between the schizophrenia probands and their relatives (Cohen’s *d*=0.61)^22^ is consistent with prior research indicating that social anhedonia is a sensitive trait that genetically correlates with schizophrenia.^2,23^

In particular, our previous Chinese data^20^ showed that the schizotypy group reported higher levels of RSAS than the non-schizotypy group, and the patient group reported higher levels of RSAS than the schizotypy group. However, the schizotypy group did not differ from the schizophrenia group in terms of RSAS scores, although these two groups differed significantly from the non-schizotypy group. These data add to the growing body of literature, suggesting that social anhedonia, decreased pleasure experiences in the social environment, may be a valuable target for identification and early intervention in high-risk populations, as well as in clinical samples.

One of the strengths of the present study is the relatively large number of first-degree relatives of schizophrenia patients; indeed, with the exception of the Roscommon study,^24^ this may be one of the largest samples that has been examined for schizotypal characteristics. A particular strength of the present investigation is our inclusion of older adults. A frequent criticism of the psychometric high-risk paradigm is that it relies heavily on the study of young adult samples. The sample assessed in the present investigation is a diverse one, in terms of age. Due to the one-child rule that was present during the recruitment period and throughout the lives of many of the participants in the sample, we were unable to recruit any siblings in the relatives group.

A possible limitation of this investigation is that it is based solely on self-report measures. Some might assume that reliance on self-report information might affect the validity of the data, through over- or under-reporting of pathological symptoms and experiences. However, particularly in the case of sensitive and potentially stigmatizing conditions such as schizophrenia and related conditions, the extant data suggest otherwise.^12^ Nonetheless, further corroboration of schizotypal status using other validated methods, including, but not limited to biobehavioral measures, would enhance further studies in this area.

In summary, our data provide evidence for structural invariance in the CPPS across Chinese samples of patients, relatives, and non-clinical adults. This is an important finding, in that it supports the suitability of the CPPS, one of the most widely used questionnaire measures in schizotypy research, for cross-cultural and/or genetic investigations of schizotypy.

## Materials and methods

### Participants

One hundred and ninety six patients with schizophrenia were recruited from the Community Health Service Centre of the Chaoyang District, Beijing. All patients met the diagnostic criteria for schizophrenia according to the DSM-IV.^25^ The patients’ mean age of onset was 24.67 (±8.71) years, with a mean illness duration of 18.50 (±10.1) years. All patients were receiving anti-psychotic medication at the time of assessment, and the mean anti-psychotic dosage was 304.57 mg per day (s.d.=269.68) in chlorpromazine equivalents.^26,27^

One hundred and ninety seven non-psychotic first-degree relatives were recruited from the same site as the patient sample. All of the recruited relatives were parents of the schizophrenia patients. Their ages ranged from 28 to 79, with the mean age of 61.72 (±16.20) years. Slightly less than half (48%) of the relatives were over age 65: 57 were under age 55, 45 were between 55 and 65 years old, and the rest were over age 66. The three relative groups did not differ in terms of gender breakdown, or Chapman scale scores. However, the youngest group of relatives had more years of education than the others (*P*<0.01).

Other participants including 1,724 college students were recruited from three universities in Beijing, Shanghai and Guangzhou. The student sample, which was reported previously,^13^ theoretically included psychometric schizotypes, i.e., those individuals whose schizotypal status might be identified on the basis of their aberrant questionnaire response profiles.

### Materials

The current study used similar methods as a previous investigation.^13^ In brief, we administered a set of questionnaires, including four CPPS intermixed with an infrequency scale to detect invalid or random responses to all the participants to capture schizotypal traits.

Validated Chinese translations of the four scales^28^ were used. The 61-item RPAS^29^ was designed to measure a deficit in the ability to experience sensory and aesthetic pleasure. The 40-item RSAS^30^ was designed to assess social withdrawal and deficits in the ability to experience pleasure from social and/or interpersonal relationships. The 35-item PER^31^ measures transient body image and perceptual distortions, with items such as “I have sometimes felt that some part of my body no longer belongs to me” (keyed true). The 30-item MIS^32^ assesses belief in causality that is not valid (e.g., “Good luck charms don’t work”; keyed false). Information regarding the psychometric properties of the CPPS can be found elsewhere.^33,34^

### Procedures

This study was approved by the Ethics Committee of the Institute of Psychology, the Chinese Academy of Sciences. Written informed consent was obtained from all the participants before the administration of the questionnaires. The college students completed the questionnaires in groups of ~60–100 students. For patients with schizophrenia and their non-psychotic first-degree relatives, the questionnaires were administered individually by trained psychology graduates.

### Data analysis

Confirmatory factor analysis (CFA) and measurement invariance models were conducted to examine the invariance structure across samples. Due to the large number of items in each of the Chapman scales, the items of the four scales were divided into three “parcels”, according to Little *et al.*^35^ to yield more robust estimates for subsequent CFAs. Readers are referred to Chan *et al.*^13^ for a description of the parceling procedure. The resultant parcels included a balanced proportion of items from each third of the scale. Cronbach’s *α* coefficients^36^ were computed to determine the internal consistency of each of the Chapman scales.

We used the optimal model for Chinese samples, which was confirmed by Chan *et al.*^13^ as the default model (see Figure 1). This model is a variation of the model first tested in the previous work of Kwapil *et al*.^37^ The model is a two-factor model in which there is a positive schizotypy factor (with loadings from the PER and the MIS parcels) and a negative schizotypy factor (with loadings from the RSAS and the RPAS parcels), whereby the items in the MIS and the PER are correlated independently.

The fitness of models in each samples were conducted individually, followed by the cross-sample invariance analysis. Goodness-of-fit was assessed using multiple indicators, including the CFI, the TLI (non-normed fit index) and its confidence interval, the RMSEA, and the *χ*^2^-statistic. However, absolute indices were used to evaluate the overall model fit because the *χ*^2^-tests were sensitive to sample size and might yield misleading findings.^38^ Therefore, we also evaluated the goodness-of-fit using the SRMR, the Akaike information criterion and the Bayesian Information Criterion (BIC). The CFA and invariance analysis were conducted in Mplus.^39,40^ Finally, a series of one-way analysis of variances were conducted to compare the schizophrenia patients, relatives, and students in terms of their mean CPPS scores across the three samples.

We evaluated model invariance across the three different groups of participants by conducting a series of invariance tests according to Marsh *et al*.^41^ All the tested models are listed in Table 3. These models were partially nested; the models differed in terms of their level of restrictiveness and the parameters that were constrained. Model 1, the least restrictive model, only constrained the factorial structure. Model 1 is the first step to establishing measurement invariance. Model 2 added the constraint of equal factor loadings. Model 3 constrained factor loadings, as well as equal item intercepts. Model 4 added the constraint of error invariance. Model 5 was nested under Model 3, meaning that in addition to all of the constraints in Model 3, Model 5 also required equal factor variance—covariances across the groups. Model 6, which was nested under Model 5, added factor means. Model 7 was nested under Model 2, thereby constraining the factor loadings and the factor variance—covariances. Model 8 was nested under Model 7, so factor means, factor loadings, and factor variance—covariances were all constrained.

## Figures and Tables

**Table 1:** Comparison of demographic data and Chapman scale scores for schizophrenia patients, first-degree relatives, and college students

*Variable*	*Schizophrenia patients (*n*=196) mean (s.d.)*	*First-degree relatives (*n*=197) mean (s.d.)*	*College students (*n*=1724) mean (s.d.)*	χ^*2*^ */F*	P
Gender (male/female)	101/95	84/113	645/1079	15.79	<0.001
Age (years)	43.31 (9.61)	61.72 (16.2)	18.81 (0.83)	5,916.53	<0.001
Education (years)	11.64 (2.62)	10.73 (3.57)	12.31 (0.73)	105.38	<0.001
PER	9.36 (7.95)	7.25 (7.69)	6.93 (5.85)	13.27	<0.001
MIS	10.87 (5.80)	8.72 (5.24)	12.01 (4.73)	42.46	<0.001
RSAS	16.22 (6.88)	12.20 (6.19)	8.17 (4.42)	277.74	<0.001
RPAS	24.74 (10.30)	21.46 (8.40)	15.45 (7.26)	166.70	<0.001

Abbreviations: MIS, magical ideation scale; PER, perceptual aberration; RPAS, revised physical anhedonia scale; RSAS, revised social anhedonia scale.

Higher scores indicate greater levels of the schizotypal trait.

**Table 2:** The model fitness indicators in three samples

*Model*	χ^*2*^	*df*	P	*CFI*	*TLI*	*RMSEA*	*CI90*		P *values*	*SRMR*
Patients with schizophrenia	104.826	47	<0.0001	0.964	0.949	0.079	0.059	0.100	0.011	0.071
First-degree relatives	121.993	47	<0.0001	0.954	0.935	0.09	0.071	0.110	0.001	0.072
College students	332.336	47	<0.0001	0.971	0.96	0.059	0.053	0.065	0.005	0.047

Abbreviations: CFI, comparative fit index; df, degree of freedom; RMSEA, root mean square error of approximation; SRMR, standardized root mean square residual; TLI, Tucker–Lewis index.

Goodness-of-fit of the model for schizophrenia patients, first-degree relatives, and college students was assessed using multiple indicators, including *χ*^2^, the CFI, TLI, RMSEA and its confidence interval, as well as the SRMR.

**Table 3:** Cross-sample test model fitness indicators

*Model no.*	*Nest*	*Parameter constrained*	*Sample*	χ^*2*^	*df*	χ^*2*^ */df*	*Δ*χ^*2*^ */Δdf*	*CFI*	*ΔCFI*	*TLI*	*ΔTLI*	*SRMR*	*RMSEA (90% CI)*	*AIC*	*BIC/adjusted BIC*
1		None		559.154	141	3.97		0.968		0.956		0.053	0.065(0.059 0.071)	102,047.98	102,777.83/102,367.983
			S	104.826											
			R	121.993											
			STU	332.336											
2	1	FL		639.86	161	3.97	4.04***	0.964	0.004	0.955	0.001	0.062	0.065(0.060 0.070)	102,088.685	102,705.381/102,359.076
			S	138.416											
			R	155.251											
			STU	346.194											
3	1, 2	FL, INT		1,060.69	181	5.86	21.04***	0.933	0.031	0.927	0.028	0.073	0.083(0.078 0.088)	102,469.515	102,973.055/102,690.293
			S	285.153											
			R	353.651											
			STU	421.886											
4	1, 2, 3	FL, INT, UNQ.		1,252.441	205	6.11	7.99***	0.921	0.012	0.923	0.004	0.078	0.085(0.081 0.090)	102,613.267	102,981.021/102,774.509
			S	354.593											
			R	387.961											
			STU	509.888											
5	1, 2, 3	FL, INT, FVCV		1,139.831	187	6.10	13.19***	0.928	0.005	0.924	0.003	0.097	0.085(0.080 0.090)	102,536.657	103,006.250\102,742.550
			S	329.744											
			R	375.36											
			STU	434.726											
6	1, 2, 3, 5	FL, UNQ, FVCV, FMn		1,558.336	191	8.16	104.63***	0.896	0.032	0.893	0.031	0.125	0.101(0.096 0.105)	102,947.161	103,394.124/103,143.133
			S	598.898											
			R	449.926											
			STU	509.512											
7	1, 2	FL, FVCV		719.409	167	4.31	13.26***	0.958	0.006	0.95	0.005	0.088	0.068(0.063 0.074)	102,156.234	102,738.983/102,411.741
			S	182.48											
			R	175.926											
			STU	361.002											
8	1, 2, 7	FL, FVCV, FMn		959.161	171	5.61	59.94***	0.94	0.018	0.931	0.019	0.097	0.081(0.076 0.086)	102,387.987	102,948.104/102,633.570
			S	331.201											
			R	243.126											
			STU	384.834											

Abbreviations: Nest, the current model nested in the previous model; Parameter(s) constrained: FL, factor loadings constrained to be equal across samples; FMn, factor mean; FVCV, factors‘ variance and covariance; INT, item intercepts; UNQ, uniqueness (error variance); Samples: S, schizophrenia patients; R, first-degree relatives; STU, college students. Goodness-of-fit indicators: CFI, comparative fit index; TLI, Tucker-Lewis index; SRMR, the Standardized Root Mean Square Residual; RMSEA, root mean square error of approximation; AIC, Akaike information criterion; BIC, Bayesian information criterion. ***=*P*<0.001.
