# Supplementary material for: Evidence of structural invariance across three groups of Meehlian schizotypes
Source: NPJ Schizophr. 2016 May 4;2:16016–. doi: 10.1038/npjschz.2016.16 (PMC4900100; doi:10.1038/npjschz.2016.16)
Supplement: Supplementary Table 1 [file npjschz201616-s1.pdf]

Supplementary Table 1. Detailed demographic data and Chapman scale scores for the first-degree relatives grouped by age.

	Under Age 55 (n = 57)	55 - 65 (n = 45)	Over Age 66 (n = 95)	χ^2/F	p
Variable	Mean (SD)	Mean (SD)	Mean (SD)		
Age (years)	41.41 (13.20)	60.53 (3.11)	74.46 (5.10)	299.54	<0.001
Gender (male/female)	22/35	16/29	46/49	2.60	n.s.
Education (years)	11.95 (3.19)	9.60 (3.03)	10.54 (3.83)	6.01	<0.01
PER	6.46 (6.88)	7.36 (8.74)	7.67 (7.69)	0.44	n.s.
MIS	8.61 (5.23)	7.98 (5.45)	9.14 (5.15)	0.77	n.s.
RSAS	11.11 (5.29)	12.69 (6.83)	12.63. (6.35)	1.26	n.s.
RPAS	20.25 (9.36)	21.96 (7.93)	21.46 (8.40)	0.57	n.s.

Note: Means and Standard deviations (SD) for each of the following Chapman psychosis-proneness scales: Perceptual Aberration (PER); Magical Ideation Scale (MIS); Revised Social Anhedonia Scale (RSAS); and Revised Physical Anhedonia Scale (RPAS). Higher scores indicate greater levels of the schizotypal trait.
