# Supplementary material for: Evidence of structural invariance across three groups of Meehlian schizotypes
Source: NPJ Schizophr. 2016 May 4;2:16016–. doi: 10.1038/npjschz.2016.16 (PMC4900100; doi:10.1038/npjschz.2016.16)
Supplement: Supplementary Table 2 [file npjschz201616-s2.pdf]

Supplementary Table 2. The reliability of the Chapman scales in three samples before and after parceling.

Sample	n	RSAS	RPAS	MIS	PER
S	196	0.83	0.89	0.83	0.92
S parceled	196	0.78	0.89	0.83	0.93
R	197	0.82	0.84	0.82	0.94
Rparceled	197	0.79	0.84	0.84	0.94
STU	1724	0.82	0.83	0.75	0.89
STUparceled	1724	0.81	0.83	0.77	0.89

Note: The reliability, as measured by Cronbach's alpha, of the Chapman psychosis-proneness scales before and after parceling for the schizophrenia patients (S), first-degree relatives (R), and college students (S).
RSAS=revised Social Anhedonia Scale; RPAS= Revised Physical Anhedonia Scale; MIS= Magical Ideation Scale; PER= Perceptual Aberration Scale.
